# Hexokinase-2-mediated aerobic glycolysis is integral to cerebellar neurogenesis and pathogenesis of medulloblastoma

**DOI:** 10.1186/2049-3002-1-2

**Published:** 2013-01-23

**Authors:** Timothy R Gershon, Andrew J Crowther, Andrey Tikunov, Idoia Garcia, Ryan Annis, Hong Yuan, C Ryan Miller, Jeffrey Macdonald, James Olson, Mohanish Deshmukh

**Affiliations:** 1 Department of Neurology, University of North Carolina, Chapel Hill, NC, 27599, USA; 2 Neuroscience Center, University of North Carolina, Chapel Hill, NC, 27599, USA; 3 Lineberger Comprehensive Cancer Center, University of North Carolina, Chapel Hill, NC, 27599, USA; 4 Joint Department of Biomedical Engineering, NC State University and UNC Chapel Hill, Chapel Hill, NC, 27599, USA; 5 Department of Cell and Developmental Biology, University of North Carolina, Chapel Hill, NC, 27599, USA; 6 Department of Radiology, University of North Carolina, Chapel Hill, NC, 27599, USA; 7 Department of Pathology, Division of Neuropathology, University of North Carolina, Chapel Hill, NC, 27599, USA; 8 Clinical Research Division, Fred Hutchinson Cancer Research Center, Seattle, WA, 98109, USA; 9 UNC School of Medicine, 170 Manning Drive CB7025, Chapel Hill, NC, 27599, USA

**Keywords:** Warburg effect, Aerobic glycolysis, Medulloblastoma, Smoothened, Brain tumor, Cerebellum

## Abstract

**Background:**

While aerobic glycolysis is linked to unconstrained proliferation in cancer, less is known about its physiological role. Why this metabolic program that promotes tumor growth is preserved in the genome has thus been unresolved. We tested the hypothesis that aerobic glycolysis derives from developmental processes that regulate rapid proliferation.

**Methods:**

We performed an integrated analysis of metabolism and gene expression in cerebellar granule neuron progenitors (CGNPs) with and without Sonic Hedgehog (Shh), their endogenous mitogen. Because our analysis highlighted Hexokinase-2 (Hk2) as a key metabolic regulator induced by Shh, we studied the effect of conditional genetic Hk2 deletion in CGNP development. We then crossed Hk2 conditional knockout mice with transgenic SmoM2 mice that develop spontaneous medulloblastoma and determined changes in SmoM2-driven tumorigenesis.

**Results:**

We show that Shh and phosphoinositide 3-kinase (PI3K) signaling combine to induce an Hk2-dependent glycolytic phenotype in CGNPs. This phenotype is recapitulated in medulloblastoma, a malignant tumor of CGNP origin. Importantly, cre-mediated ablation of Hk2 abrogated aerobic glycolysis, disrupting CGNP development and Smoothened-induced tumorigenesis. Comparing tumorigenesis in medulloblastoma-prone SmoM2 mice with and without functional Hk2, we demonstrate that loss of aerobic glycolysis reduces the aggressiveness of medulloblastoma, causing tumors to grow as indolent lesions and allowing long-term survival of tumor bearing mice.

**Conclusions:**

Our investigations demonstrate that aerobic glycolysis in cancer derives from developmental mechanisms that persist in tumorigenesis. Moreover, we demonstrate in a primary tumor model the anti-cancer potential of blocking aerobic glycolysis by targeting Hk2.

See commentary article:http://www.biomedcentral.com/1741-7007/11/3

## Background

Aerobic glycolysis, the metabolism of glucose to lactate despite the availability of oxygen, is observed in diverse cancers, a phenomenon known as the Warburg effect [1,2]. Indeed, many cancers, including brain tumors, demonstrate increased glucose utilization, suggesting that glycolytic metabolism may confer a selective advantage [3,4]. Less is known about metabolic adaptations during development. Examining these adaptations is important because metabolic patterns that support cancerous growth may derive from genetic programs that evolved to support developmental growth.

Neurogenesis, like tumorigenesis, requires rapid cellular proliferation, but under precise control. In human brain development, over 80 billion cerebellar granule neurons (CGNs) are generated in the first 6 months of life. Many of the developmental milestones observed in the first year of life are directly attributed to proper formation of cerebellar neural circuits involving the granule neurons. Excessive proliferation and retarded maturation of CGNPs, often driven by mutations in neurodevelopmental genes, give rise to medulloblastoma, the most common malignant brain tumor in children [5,6]. We hypothesized that aerobic glycolysis is integral to the regulated proliferation of neural progenitors, and that aerobic glycolysis in cancer may result from the abnormal persistence of metabolic programs that are typically restricted to development. We therefore investigated the relationship between glucose metabolism and neural progenitor function during cerebellar development and medulloblastoma pathogenesis.

Postnatal neurogenesis in the cerebellum presents an ideal opportunity to study metabolic dynamics of neurogenesis under aerobic conditions. CGNs are the most numerous cells in the brain, and arise from CGNPs that proliferate in the external granule cell layer (EGL) in a wave of neurogenesis that occurs postnatally and lasts until postnatal day (P) 15 in mice [7]. CGNPs thus proliferate under normoxic conditions, and mouse cerebellum may be sampled at defined time points to include proliferating neural progenitors or exclusively post-mitotic neurons. As CGNPs terminally differentiate, they migrate from the EGL to the internal granule cell layer (IGL) such that position in the cerebellum corresponds with differentiation state. CGNPs are readily cultured and maintain their proliferative behavior *in vitro* in serum-free media supplemented with Shh and insulin [8,9]. If Shh is withdrawn, CGNPs exit the cell cycle and differentiate – such that after 24 hours in culture without Shh, proliferation is minimal. Importantly, activating mutations in the Shh pathway have been found in human medulloblastoma and can recapitulate tumorigenesis in transgenic mice, including the ND2:SmoA1 and SmoM2 lines that express constitutively active alleles of Smoothened [5,10-13]. These animal models consistently implicate CGNPs as proximal cells of origin for Shh-driven medulloblastoma. Here, we examine glucose metabolism in CGNPs, CGNs and Smoothened-induced, murine medulloblastomas in order to determine whether aerobic glycolysis originates in neural development and whether this metabolic pattern is essential to the pathogenesis of embryonal cancers of the nervous system.

## Methods

### Animals

Mice were handled in compliance with the guidelines of the University of North Carolina Animal Care and Use Committee. NeuroD2:SmoA1 mice were provided by Dr James Olson (Fred Hutchinson Cancer Research Center, Seattle, WA, USA) and SmoM2 mice (Bl6 background) were purchased from Jackson Laboratories (Bar Harbor, ME, USA). hGFAP-cre mice were generously provided by Dr Eva Anton (University of North Carolina, Chapel Hill, NC, USA); these mice were initially obtained in the FVB/N background, and were crossed into the Bl6 background at least 10 times. Hk2^fl/fl^ mice were obtained from the European Mouse Mutant Archive and are documented on the archive’s website. In brief, these mice (deposited by Dr Eija Pirinen) harbor LoxP sites at intron 3 and intron 10 of the Hk2 gene, such that exons 4 to 10 are deleted in the presence of cre recombinase. Hk2^fl/fl^ mice were crossed at least 5 times with Bl6 mice prior to the experimental breeding. Medulloblastomas were detected by daily observation for abnormalities of head shape and movement, and animals were sacrificed at the onset of tumor symptoms, specifically ataxia, weight loss or movement disorder. For EdU experiments, mouse pups at P10 were injected intraperitoneally (IP) with 50 μl HBSS containing EdU (250 μM; catalogue number A10044; Life Technologies, Grand Island, NY, USA) and sacrificed after 24 hours. All animal handling and protocols were carried out in accordance with established practices as described in the National Institutes of Health Guide for Care and Use of Laboratory Animals and as approved by the Animal Care and Use Committee of the University of North Carolina (IACUC #10-126.0).

### Cell culture techniques

**Figure 1:**
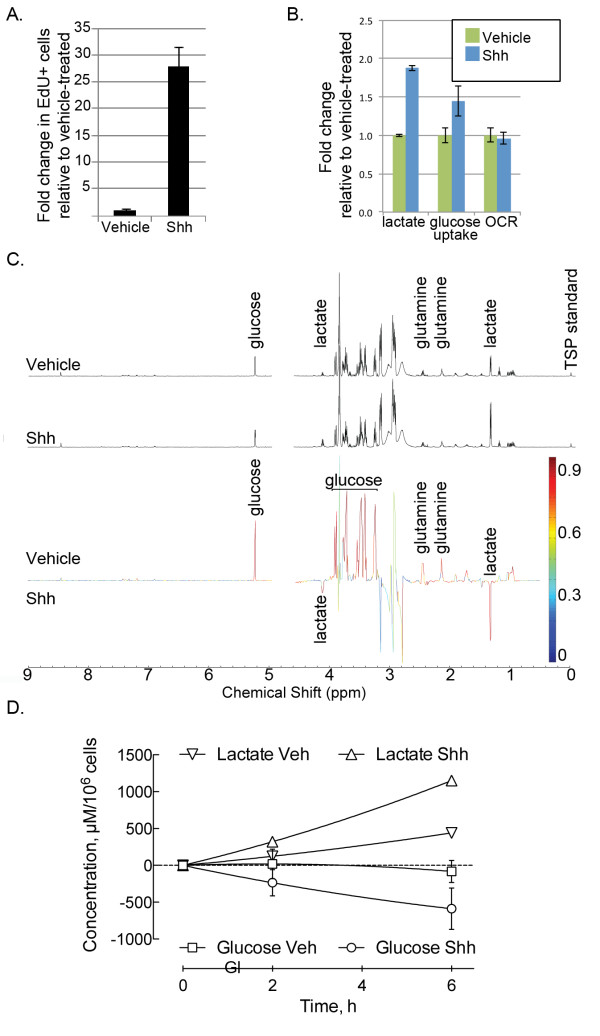
**Shh induces aerobic glycolysis in CGNPs. (A)** Counts of EdU^+^ cells, in 3 replicate wells for each condition, confirm that Shh-treated CGNPs continue proliferation after 48 hours in culture, while vehicle-treated CGNPs exit the cell cycle. **(B)** Lactate production, glucose uptake and oxygen consumption rate (OCR) of Shh-treated and vehicle-treated CGNPs are compared, using 3 replicate wells per condition. Measured values were normalized for cell number and expressed as fold-change relative to vehicle-treated values. Shh increased lactate production (*P* < 0.01) and glucose uptake (*P* < 0.03) while no statistically significant effect on the OCR was detected. **(C)** NMR spectra (representative examples on top; below is orthogonal partial least squares discriminant analysis comparison of 6 replicates of each condition) demonstrate differential accumulation of lactate in media of Shh-treated CGNPs compared with vehicle-treated CGNPs. The loading coefficient is plotted as the *y* value, and the *P* scaled correlation coefficient is color-coded as indicated. Lactate peaks are deflected toward Shh, indicating greater value in Shh-treated wells, and color-coded red, indicating statistical significance. **(D)** Starting with fresh media at time 0, Shh-treated CGNPs used more glucose (*P* < 0.001) and produced more lactate (*P* < 0.001) than vehicle-treated CGNPs over a 6-hour period. Importantly, in Shh-treated CGNPs, glucose utilization and lactate production were in a stoichiometric 1:2 ratio. Graphs present mean ± standard error of the mean (SEM). Two-tailed Student’s *t* test was used for statistical comparisons in (**A**) and (**B**), while two-way analysis of variance with Bonferroni correction was used in (**D**).

CGNP cultures were generated as previously described [14]. Briefly, cerebella were dissected from P5 mouse pups, dissociated, and allowed to adhere to culture wells in DMEM/F12 (catalogue number 11320; Life Technologies, Grand Island, NY, USA) with 25 mM or 4 mM KCl as indicated, supplemented with N2 and 5% FCS for 4 hours, after which media were replaced with identical, serum-free media. For 5.6 mM glucose experiments, DMEM/F12 was replaced with DMEM low glucose (catalogue number 11885; Life Technologies, Grand Island, NY, USA) supplemented with N2 and KCl to 25 mM. Media were replaced every 24 hours with fresh media. Shh-treated CGNPs were maintained continuously in Shh (0.5 μg/ml, catalogue number 464SH; R&D Systems Minneapolis, MN, USA). For hypoxia studies, CGNPs were plated under normoxic conditions and allowed to adhere overnight in media supplemented with Shh and N2. Media were then replaced with media that was preconditioned in a 2% O_2_ incubator and supplemented with Shh and N2 as indicated. CGNPs were then maintained in a 2% O_2_ incubator for 24 hours, after which lysates were rapidly prepared under normoxia. Where indicated, Myc inhibitor 10058-F4 (catalogue number 475956; Calbiochem San Diego, CA, USA) was added to cultures after the first 24 hours, at the concentrations specified, and cells were harvested 24 hours later. All metabolic measurements were performed on 3 replicate wells for each condition, except for the NMR studies in Figure 1C,D in which 6 replicates were used. Cell counts were performed at the end of each experiment in order to normalize for the number of cells per well. For cell counts, cells were incubated with 1 mM bisbenzimide for 30 minutes, photographed through a 20× objective and nuclei were counted using Leica-Metamorph software (Molecular Devices Sunnyvale, CA, USA). 

### *In vitro* metabolism studies

For enzymatic measurement of lactate, media were sampled after 48 hours in culture and lactate was quantified by the l-Lactate Assay Kit (catalogue number 1200011002; Eton Bioscience Durham, NC, USA) using the manufacturer’s protocol. For 18-fluorodeoxiglucose (^18^FDG) studies, CGNPs were cultured for 48 hours, incubated for 40 minutes in 2 μCi ^18^FDG in glucose-containing DMEM/F12 supplemented as indicated, washed twice, and collected. The concentration of FDG was less than 0.1 nM, and control experiments in which Shh-treated CGNPs were treated with either normal media or with media containing 1 nM 2-deoxyglucose demonstrated no change in lactate production or CGNP proliferation, measured by incorporation of EdU (data not shown). Radioactivity was measured by gamma counter (2470 Wizard2; PerkinElmer Waltham, MA, USA) and normalized to the activity measured in the initial media. For oxygen consumption rate (OCR) measurements, after 48 hours in culture with either vehicle or Shh, CGNPs were changed to fresh media and the OCR was measured using a Seahorse XF24 (Seahorse Bioscience, North Billerica, MA, USA) following the manufacturer’s protocol. The electron transport uncoupling agent trifluorocarbonylcyanide phenylhydrazone (FCCP; 300 nM) was added, and OCR measurements were then repeated immediately. For proliferation assays, EdU was added to the cell culture and visualized using the manufacturer’s protocol (catalogue number C10337; Life Sciences), and positive cells were counted using Leica-Metamorph software (Molecular Devices). For Hk activity assays, cells from 3 replicate wells per condition, or snap-frozen cerebella from 3 replicate mice per genotype, were lysed and processed for colorimetric assay per manufacturer’s protocol (Hexokinase Assay Kit, catalogue number E-111; Biomedical Research Service Center, SUNY, Buffalo, NY, USA).

For NMR-based metabolomic analysis, cells were plated in 12-well plates in 650 μl media and then 50 μl media samples were harvested at the indicated time points. Cell counts on the day of media harvest demonstrated that all wells contained 95 to 105% of the mean number of cells, and there was no statistically significant variation in cell number in wells treated with Shh or vehicle (data not shown). Media samples were processed as previously described [15]. Briefly, proton (^1^H) spectra were acquired at 25°C on a 14.1 T Varian INOVA spectrometer (600 MHz ^1^H frequency) equipped with a CapNMR™ microcoil (Magnetic Resonance Microsensors Corp, Savoy, MN, USA). The ^1^H spectra were acquired using a one-pulse sequence with presaturation of the water resonance using a 90° flip angle, and a total repetition time of 12.65 seconds. The peak areas in the ^1^H spectra were determined using Chenomx NMR processing software version 7.1 (Edmonton, Alberta, Canada). First, spectra were zero-filled to 32,000 points, and were line broadened using a 0.5 Hz exponential Gaussian function. Chemical shifts presented were obtained from the Human Metabolome Database [16]. Concentrations were calculated from the ^1^H spectra by comparing peak areas with the peak for 2,2^′^,3,3^′^-duetero-trimethyl propionate. Concentration values were then normalized for the cell number in each well, and the results were analyzed by two-way analysis of variance with Bonferroni correction. For statistical comparison of multiple spectra, we performed orthogonal partial least squares discriminant analysis using ACD Labs 12.0 1D NMR Processor (ACD Labs Toronto, Ontario, Canada) to zero-fill to 32,000 points, with a 0.5 Hz exponential Gaussian function applied, then spectra were binned into 0.005 ppm segments and values were exported to SIMCA-P + 11 (Umetrics Umeå, Sweden). Loading coefficients and *P-*scaled correlation coefficients were exported to MatLab (Mathworks, Natick, MA, USA) and plotted as the *y* value (loading coefficient) and color coded (correlation coefficient).

### *In vivo* metabolism studies

To measure cerebellar glucose uptake, mouse pups at P5 or P20 were injected IP with 0.2 mCi ^18^FDG; after 40 minutes, pups were rapidly decapitated and the cerebella and forebrain were harvested by dissection. Tissue samples were washed and weighed, and incorporated radioactivity was quantified by gamma counter (2470 Wizard2; PerkinElmer). Incorporated counts from the cerebellum were normalized for tissue weight and for dose to the brain, as measured by incorporated radioactivity in the frontal lobe sample from the same animal.

Magnetic resonance spectroscopy (MRS) acquisitions were performed at 9.4 T on a Bruker BioSpec 94/30 MRI system (Bruker BioSpin, Bilerica, MA, USA). A volume of interest was placed on the pup cerebellum region based on T2-weighted images with a size of 11.5 mm^3^. A point-resolved spectroscopy sequence was used for single-voxel signal acquisition (Echo Time = 1.4 ms; total repetition time = 20,000 ms; 64 × 64 matrix size). The spectrum was adjusted with the water signal at 4.7 ppm as a reference.

^18^FDG positron emission tomography/computed tomography (PET/CT) imaging was performed on a PET/CT scanner (GE eXplore Vista PET/CT; GE Helathcare Worldwide, Waukesha, WI, USA). Under isoflurane anesthesia, mice underwent intravenous administration of 500 μCi ^18^FDG and computed tomography scan. Thirty minutes after ^18^FDG injection, PET/CT imaging was acquired over 10 minutes. Images were reconstructed using ordered subset expectation maximization algorithms, and were normalized to dose and animal weight to generate standardized uptake values of the final images.

### Histology and immunohistochemistry

Mouse brain and tumor tissue were embedded in paraffin and sectioned to 5 μm thickness. H & E-stained sections were prepared using standard techniques. EdU was detected using the Click-iT® EdU Alexa Fluor 488 Imaging Kit (catalogue number C10337; Life Sciences), as per the manufacturer’s protocol. Immunohistochemistry (IHC) was performed on paraffin-embedded sections after deparaffinization in Histoclear, rehydration in a graded ethanol series, and antigen retrieval by heating to boiling in 10 mM citrate buffer pH 6.0 in a pressure cooker for 15 minutes and then transferring to PBS. For Hk2 detection, tissue was not embedded in paraffin but rather was sectioned by Vibratome to 100 μm thickness and stained by IHC without antigen retrieval. IHC was performed as previously described using primary antibodies: Hk1 (catalogue number 2024; Cell Signaling Technologies, Danvers, MA, USA), GFP (catalogue number 600-101-215; Rockland Immunochemicals, Gilbertsville, PA, USA), Hk2 (catalogue number 2867; Cell Signaling), Calbindin (catalogue number 2173; Cell Signaling), CD31 (catalogue number 3528 Cell Signaling), NeuN (catalogue number MAB377; Millipore, Billerica, MA, USA), proliferating cell nuclear antigen (PCNA, catalogue number 2586; Cell Signaling), and p27 (catalogue number 3686; Cell Signaling). After EdU and IHC staining, nuclei were counterstained with 4^′^6-diamino-2-phenylindole (DAPI; catalogue number D1306; Life Sciences), diluted 200 ng/ml in PBS for 5 minutes, and immunoreactivity was evaluated with a Leica epifluorescence DM5000B microscope (Leica Microsystems, Wetzlar, Germany). Stained slides were then scanned using an Aperio ScanScope XT (Vista, CA, USA).

### Western blot analysis

Cultured cells, whole cerebella, and tumors were lysed by homogenization in lysis buffer (catalogue number 9803; Cell Signaling). Protein concentrations were quantified using the Bicinchoninic acid method (catalogue number 23227; Thermo Scientific Asheville, NC, USA) and equal concentrations of protein were resolved on SDS-polyacrylamide gels then transferred to polyvinylidene fluoride membranes. Immunologic analysis was performed on a SNAP ID device (Millipore) using the manufacturer’s protocol with primary antibodies to β-actin (catalogue number 4970; Cell Signaling), Hk1 (catalogue number 2024; Cell Signaling), Hk2 (catalogue number 2867; Cell Signaling), Cyclin D2 (catalogue number 3741; Cell Signaling), insulin-like growth factor (IGF) receptor (catalogue number 9750; Cell Signaling), phospho-IGF receptor (catalogue number 6113; Cell Signaling), Akt (catalogue number 4685; Cell Signaling), pAkt (catalogue number 4060; Cell Signaling), HP-Hif1a (catalogue number 3434; Cell Signaling), phospho-AMP-activated kinase (catalogue number 2535; Cell Signaling), phospho-Acyl-CoA carboxylase (catalogue number 3661; Cell Signaling), caspase-3 (cC3, catalogue number 9664; Cell Signaling), GFP (catalogue number 600-101-215; Rockland), Smo (catalogue number AB72130; Abcam, Cambridge, MA, USA), and Cip2A (catalogue number SC-80660; Santa Cruz Biotechnology Santa Cruz, CA, USA). Secondary antibodies were anti-rabbit IgG horseradish peroxidase (catalogue number 7074; Cell Signaling), and anti-mouse IgG horseradish peroxidase (catalogue number 7076; Cell Signaling). Antibody conjugates were visualized by chemiluminescence (catalogue number RPN2106; GE Healthcare).

### Quantitative RT-PCR

Total RNA was prepared from CGNPs using the RNeasy Mini Kit (catalogue number 74104; Qiagen, Valencia, CA) as per protocol. First-strand cDNA was synthesized using the Invitrogen SuperScript III Kit (catalogue number 18080-051, Life Sciences). To prevent amplification from genomic DNA, PCR primers were designed to span at least one intron, and PCR products were cloned and sequenced to verify identity. The PCR primers were: Hk2, ATTGTCCAGTGCATCGCGGA and AGGTCAAACTCCTCTCGCCG; Cyclin D2, GCGTGCAGAAGGACATCCA and CACTTTTGTTCCTCACAGACCTCTAG; and β-actin, ATGCTCTCCCTCACGCCATC and CAGGATTCCATACCCAAGA. PCR reactions were run on an ABI 7500Fast instrument, using ABI Fast Sybr Green master mix (catalogue number 4385612; Applied Biosystems Carlsbad, CA, USA), cycling between 95 and 60°C, as per the manufacturer’s protocol, for 50 cycles. The threshold cycle (CT) was determined by ABI proprietary software. PCR efficiency for each primer pair was measured by amplifying a series of copy number standards from cloned, sequenced PCR products and used to calculate the fold-change, using β-actin as the reference standard [17].

## Results

### Shh signaling induces aerobic glycolysis in CGNPs

To determine whether mitogenic signaling alters the glucose metabolism of neural progenitors, we compared lactate generation, glucose uptake and oxygen consumption of CGNPs cultured in the presence or absence of Shh. We isolated CGNPs from P5 mouse pups and cultured them in serum-free, N2-supplemented media, with Shh or vehicle as indicated. After 48 hours in culture, only Shh-treated CGNPs continued to proliferate (Figure 1A). Starting from fresh media at 24 hours, from 24 to 48 hours in culture, Shh-treated CGNPs accumulated 180% more lactate than Shh-deprived CGNPs that exited the cell cycle (Figure 1B). Shh-induced lactate production did not depend on the high glucose and K^+^ concentrations of typical CGNP media, as Shh induced comparable lactate production in CGNPs maintained in CGNP media (18 mM glucose, 25 mM KCl), DMEM/F12 (4 mM KCl) or low-glucose DMEM (5.6 mM glucose; see Additional file 1: Figure S1). Shh-treated CGNPs also demonstrated differential uptake of ^18^FDG when exposed briefly to the tracer in freshened 18 mM glucose culture media (Figure 1B). Despite increased glucose uptake and lactate production, Shh-treated CGNPs did not increase the OCR, measured as picomoles per minute in real-time by an XF Extracellular flux Analyzer (Seahorse Bioscience) and normalized for the number of cells per well. Importantly, both vehicle-treated and Shh-treated CGNPs increased the OCR briskly and equally when exposed to the respiratory chain uncoupling agent FCCP (data not shown), indicating that CGNPs were not constrained by the availability of oxygen. Taken together, these results demonstrate that Shh induced CGNPs to increase metabolism of glucose to lactate under conditions in which oxygen was not limiting.

To identify metabolic changes induced by Shh in a nonbiased approach, we used ^1^H NMR spectroscopy to measure metabolite accumulation in media of isolated CGNPs. NMR allows the simultaneous measurement of a large number of water-soluble metabolites, including products of lipid, amino acid and carbohydrate metabolism [15]. We compared media samples, taken at the indicated times after media change, from Shh-treated and vehicle-treated CGNPs beginning at 24 hours in culture. We generated NMR spectra from each of 6 replicate wells for each condition at 0, 2 and 6 hours after media change, and used orthogonal partial least squares discriminant analysis to identify metabolites that varied consistently with the presence or absence of Shh. This analysis highlighted lactate, glucose and glutamine as the predominant metabolites altered by Shh treatment (Figure 1C). We then conducted a more precise statistical analysis by subjecting concentrations of each metabolite at 0 and 6 hours in vehicle and Shh wells to two-way analysis of variance with Bonferroni correction; this analysis identified only glucose and lactate as changing with statistical significance with Shh (Table 1). These NMR data, demonstrating increased glucose utilization and lactate production induced by Shh, were consistent with data from colorimetric lactate detection and ^18^FDG studies (Figure 1B). Importantly, Shh induced a change in glucose concentration (2.3 mM; 0.5 mM/10^6^ cells) that was one-half of the change in lactate (4.6 mM, 1.0 mM/10^6^ cells), consistent with the stoichiometric relationship of 1 molecule of glucose giving rise to 2 molecules of lactate (Table 1 and Figure 1D). Shh thus exerted a potent effect on the energy metabolism of CGNPs, and the primary manifestation of this effect was the induction of aerobic glycolysis. 

**Table 1:** Concentrations of selected metabolites in Shh- or vehicle-containing CGNP media at the indicated times

	**Shh**			**Vehicle**			**ΔShh vs. ΔV**
	**0 hours (μM)**	**6 hours (μM)**	**Δ6 hours (μM)**	**0 hours (μM)**	**6 hours (μM)**	**Δ6 hours (μM)**	***P*** **value**
Acetate	109 ± 5	105 ± 2	**−4 ± 5**	111 ± 3	127 ± 7	**15 ± 8**	>0.05
Alanine	160 ± 4	394 ± 4	**234 ± 6**	165 ± 6	348 ± 13	**183 ± 15**	>0.05
Arginine	997 ± 49	1,002 ± 22	**5 ± 53**	990 ± 16	989 ± 26	**−2 ± 30**	>0.05
Glucose	196,834 ± 934	17,312 ± 328	**−2,372 ± 990**	19,449 ± 749	19,399 ± 405	**−49 ± 852**	**<0.001**
Glutamine	2,443 ± 99	2,037 ± 31	**−407 ± 103**	2,509 ± 92	2,515 ± 65	**6 ± 113**	>0.05
Glycine	223 ± 21	221 ± 6	**−2 ± 22**	215 ± 10	254 ± 8	**39 ± 13**	>0.05
Lactate	334 ± 27	4,952 ± 114	**4,618 ± 117**	299 ± 15	2,012 ± 49	**1,713 ± 51**	**<0.001**
Leucine	578 ± 29	483 ± 17	**−95 ± 33**	555 ± 23	560 ± 4	**5 ± 23**	>0.05
Threonine	461 ± 30	484 ± 37	**23 ± 47**	413 ± 14	487 ± 14	**74 ± 20**	>0.05
Valine	558 ± 14	499 ± 21	**−59 ± 24**	540 ± 22	552 ± 17	**12 ± 28**	>0.05

Concentrations of each metabolite were calculated from NMR spectra, with 6 replicate wells per condition. Data presented as mean ± SEM. For statistical analysis, two-way analysis of variance with Bonferroni correction was applied, and *P* values were calculated for the contrast of change in Shh (ΔShh) versus change in vehicle (ΔV).

**Figure 2:**
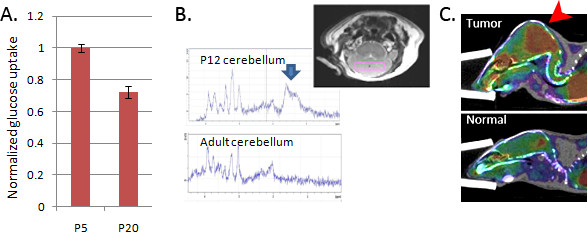
**Glycolytic phenotype is common to CGNPs and medulloblastoma *in vivo.* (A)** Cerebellar glucose flux was higher at P5, during CGNP proliferation, than at P20 after neurogenesis was completed, as measured by ^18^FDG uptake 1 hour after IP injection (*P* < 0.02). Experiment repeated 3 times using paired littermates at P5 and P20 and data presented as mean ± SEM. Uptake by forebrain was also measured and used to normalize for dose to the brain. **(B)**^1^H MRS consistently demonstrated a lactate doublet in cerebella of P12 pups (*n* = 3), in voxel defined by the box shown in the inset. This doublet was not observed in adult cerebella, or forebrain in P12 animals (data not shown). **(C)** Medulloblastoma (red arrowhead) in ND2:SmoA1 mice demonstrated markedly elevated ^18^FDG uptake on PET/CT, compared with age-matched control (below). PET/CT in 3/3 tumor-bearing mice demonstrated increased glucose uptake and a representative scan is shown.

To determine whether CGNPs utilize glucose through glycolysis *in vivo*, we compared glucose utilization and lactate production in mouse pups of various ages, either during (P1 to P15) or after (>P15) the period of CGNP proliferation. We measured cerebellar glucose uptake by injecting pups at P5 or P20 with ^18^FDG IP, harvesting the cerebella, counting incorporated radioactivity and normalizing results to tissue weight. We found 30% greater glucose uptake in P5 cerebella compared with cerebella from P20 animals (*P* < 0.02; Figure 2A). Increased glucose metabolism during the neurogenic period might be due to increased glycolysis or increased oxidative phosphorylation. To detect glycolytic activity, we measured local lactate concentration *in vivo* using ^1^H MRS. P12 pups were better suited for MRS studies than P5 pups because they are larger and still harbor proliferating CGNPs. We consistently detected lactate, identified as a doublet at 2.5 ppm, in 3/3 P12 cerebella (Figure 2B) while no lactate was detected in cerebella from adult mice (Figure 2B), or in forebrains of P12 pups (data not shown). Taken together, our *in vitro* and *in vivo* bioenergetic studies demonstrate that Shh activates a glycolytic phenotype in CGNPs that sharply contrasts the metabolic pattern of the surrounding brain.

### Glycolytic phenotype persists in medulloblastoma

Medulloblastoma cells, like CGNPs, are highly proliferative. To determine whether the high glucose flux observed in mitotic CGNPs persists in medulloblastoma, we used ^18^FDG PET/CT to compare glucose uptake in tumor-bearing and wild-type mice. We consistently detected strong glucose uptake within ND2:SmoA1-induced medulloblastomas (Figure 2C). Elevated glucose uptake in murine medulloblastoma is consistent with reported PET scan results in human medulloblastoma [18] and confirms that medulloblastomas share the glycolytic phenotype of CGNPs.

### Hk2 is induced by Shh-pathway activation and persists in medulloblastoma

Hk enzymes catalyze the first step in glucose metabolism. While there are four homologous Hk genes, Hk1 and Hk2 have been frequently associated with aerobic glycolysis [4,19]. To identify proteins that mediate the glycolytic phenotype of CGNPs and medulloblastoma, we examined the expression of Hk1 and Hk2 in CGNPs, CGNs, and ND2:SmoA1-induced medulloblastoma.

**Figure 3:**
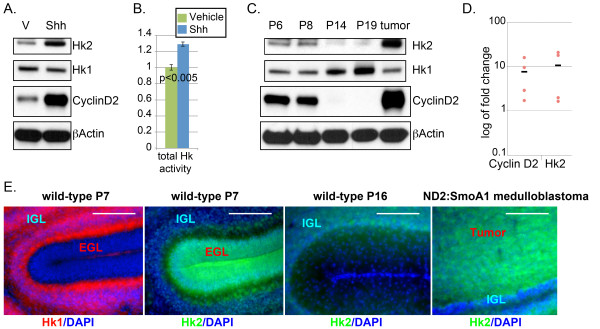
**Specific up-regulation of Hk2 in Shh-treated CGNPs and medulloblastoma. (A)** Western blot from isolated CGNPs demonstrates that exogenous Shh induces Hk2 and Cyclin D2. Hk1 expression decreased slightly with exposure to Shh. **(B)** Colorimetric assay of total Hk activity of CGNP lysates demonstrates a statistically significant increase in Hk activity in Shh-treated CGNPs. **(C)** Western blot from whole cerebella at indicated days from birth demonstrates temporal expression patterns of Hk1, Hk2 and Cyclin D2 expression. Hk2 and Cyclin D2 were strongly detected during the period of CGNP proliferation (postnatal day (P6, P8) and down-regulated by the end of cerebellar neurogenesis P14. Medulloblastoma, like mitotic CGNPs, expressed high levels of Hk2 and Cyclin D2. **(D)** Quantitative real-time RT-PCR analysis comparing mRNA expression in Shh-treated CGNPs relative to expression in vehicle-treated CGNPs. Shh induced comparable up-regulation of Hk2 and Cyclin D2. Dots indicate measured fold-change in replicate experiments, and bars indicate mean. **(E)** Immunofluorescence demonstrates reciprocal patterns of Hk1 and Hk2 at P7. CGNs of the IGL expressed Hk1, which was not detected in CGNPs of the EGL. In contrast, Hk2 was detected only in the EGL, the site of CGNPs at P7. In P16 cerebellum, where the EGL region no longer contains CGNPs, Hk2 was not detected. In medulloblastoma-bearing SmoA1 mice, Hk2 expression was widespread throughout the tumor but remained undetectable in the adjacent IGL. All scale bars = 100 μm.

We found that expression of Hk2 was induced by exposure of isolated CGNPs to Shh (Figure 3A). In contrast, expression of Hk1 was mildly reduced in Shh-treated CGNPs (Figure 3A). Consistent with the marked increase in Hk2 expression, Shh also increased the total Hk capacity of CGNPs (Figure 3B).

Previous investigations have validated Western blot for Cyclin D2 as a marker of Shh-induced proliferation [20], and we therefore compared Cyclin D2 and Hk2 in both isolated CGNPs and in whole cerebellar lysates at progressive points in postnatal development. Importantly, Hk2 expression corresponded closely with the expression of Cyclin D2 (Figure 3A,C) both with exposure to Shh *in vitro*, and *in vivo* throughout the period of postnatal neurogenesis. Hk2 and Cyclin D2 were expressed at P6 and P8, and both proteins were down-regulated by P14, as neurogenesis wanes. Hk2 and Cyclin D2 were strongly up-regulated in SmoA1-induced medulloblastoma. As with CGNPs *in vitro*, expression of Hk1 varied inversely with expression of Hk2 (Figure 3C).

To determine whether induction of Hk2 by Shh operates through transcription regulation, we compared the abundance of Hk2 mRNA in CGNPs maintained in the presence or absence of Shh. We prepared cDNA from four sets of isolated CGNPs maintained with or without Shh and measured abundance of transcripts encoding Hk2, Cyclin D2 and β-actin by quantitative real-time RT-PCR. We included Cyclin D2 as a known target of Shh-signaling and β-actin as a loading control. Shh increased expression of Hk2, driving a fold-change of 10.7 ± 2.6 (mean ± SEM), comparable with the fold increase for Cyclin D2 of 7.5 ± 1.7 (Figure 3D).

To identify the specific cells expressing Hk1 and Hk2, we examined cerebellar sections using IHC. In the P7 cerebellum, differentiated CGNs residing in the IGL uniformly expressed Hk1, while the Hk1 protein was undetectable in the entire population of CGNPs throughout the EGL (Figure 3E, P7 Hk1 panel). In contrast, Hk2 was expressed evenly throughout the EGL during neurogenesis (Figure 3E, P7 Hk2 panel). Hk2 was absent from the region of the EGL after the CGNP population had completely migrated to the IGL (Figure 3E, P16 panel), indicating Hk2-expressing cells were specifically the CGNPs. Importantly, with the development of medulloblastoma, Hk2 expression resumed (Figure 3E, medulloblastoma panel). Thus, while undifferentiated cells including CGNPs and medulloblastoma expressed Hk2, Hk1 was expressed by their differentiated progeny.

**Figure 4:**
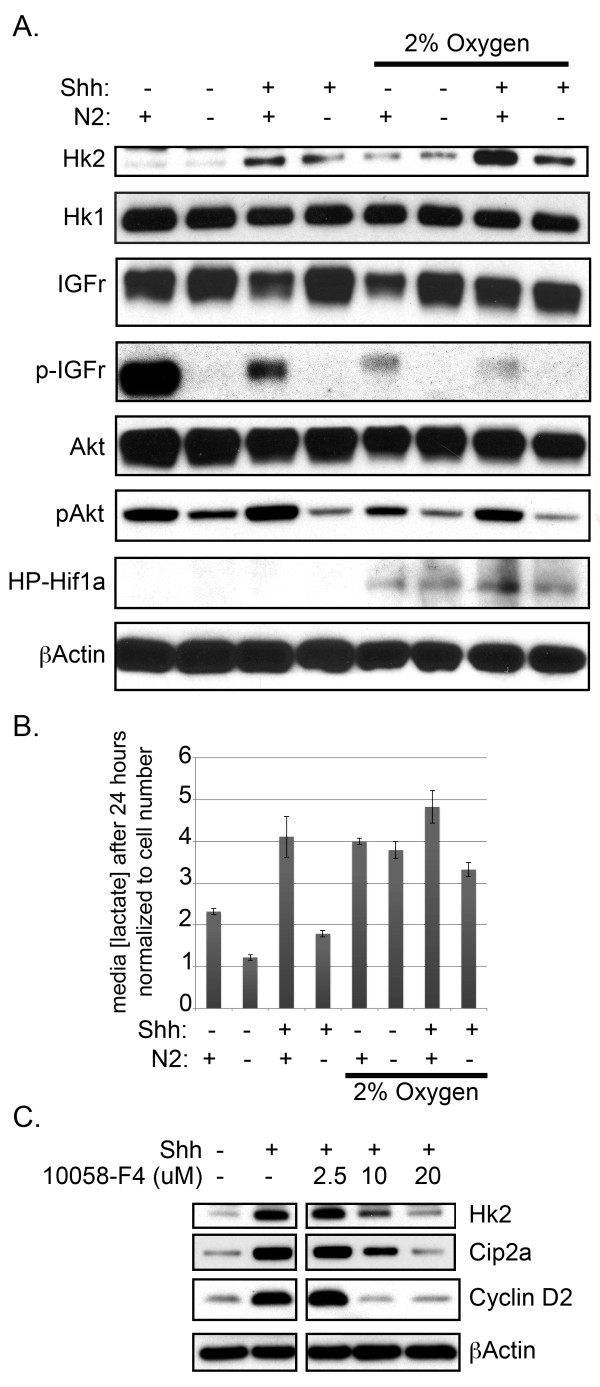
**Shh-induced expression of Hk2 and concurrent activation of glycolysis.** Shh and insulin/ IGF/ PI3K signaling pathways converge on Myc–Max effector complex to induce Hk2 expression and glycolysis. **(A), (B)** Isolated CGNPs were maintained in media with N2, Shh, neither or both. Media were changed after 24 hours in culture, after which 3 replicates per condition were maintained in normoxia for 24 hours, while 3 replicates per condition were concurrently subjected to hypoxia. Expression of Hk2, Hk1, IGFr, pIGFr, Akt, pAkt, and HP-Hif1α were demonstrated by Western blot (**A**), and the lactate concentration in media was quantified by enzymatic assay, presented as mean ± SEM, normalized for cell number (**B**). Addition of N2 alone increased Akt phosphorylation and mildly increased lactate production without inducing Hk2. Shh alone caused a modest increase in both Hk2 and lactate production. The combination of Shh and N2, however, markedly increased Hk2 expression and lactate production, indicating robust induction of glycolysis. Hypoxia alone induced near-maximal lactate production in the absence of Shh and N2, while also inducing moderate Hk2. Addition of Shh alone or N2 alone to hypoxic CGNPs did not further increase lactate, but the combination of Shh and N2 added to hypoxic CGNPs further increased both Hk2 and lactate. **(C)** Western blot analysis demonstrates that induction of Hk2 was modulated by Myc inhibitor 10058-F4 in isolated CGNPs maintained in Shh and N2. Reduced induction of Hk2 was dose dependent and paralleled the expression of Cyclin D2 and of Cip2a, a protein previously identified as down-regulated by 10058-F4.

We next examined whether the Shh-induced expression of Hk2 and concurrent activation of glycolysis were mediated by mechanisms distinct from previously described molecular regulators of metabolism. Previous investigations have demonstrated induction of Hk2 by Hif1α [21-23] and PI3K signaling [24]. To modulate Hif1α activity, we cultured CGNPs in normoxic or hypoxic conditions. To modulate activity of PI3K, we included or withheld insulin from culture media. CGNPs are typically cultured with insulin-rich N2 supplement in order to promote survival by activating P13K signaling mediated through the insulin receptor and the IGF receptor (IGFr) [9]. Previous work has demonstrated that 24 hours of N2 deprivation effectively blocks activation of the PI3K pathway in CGNPs without impairing viability [25]. By providing or withholding the ligands Shh and insulin, we were thus able to modulate the Shh and PI3K pathways without inhibitors. We exposed CGNPs to vehicle or Shh, with or without N2, under normoxic or hypoxic conditions, and then measured Hk2 protein and lactate accumulation after 24 hours (Figure 4A,B). Induction of Hif1α was confirmed by the detection of the breakdown product hydroxyprolyl-Hif1α (HP-Hif1α), and down-regulation of PI3K activity was confirmed by decreased phospho-IGF receptor (pIGFr) and decreased phospho-Akt (Figure 4A). In normoxic conditions, Shh without N2 induced a moderate increase in Hk2, with only a small increase in lactate production. N2 without Shh caused a small increase in lactate but did not induce Hk2 protein. The combination of Shh and N2, however, up-regulated Hk2 more than Shh alone, and increased media lactate with a greater than additive effect. These data indicate that, in normoxia, the combination of N2 and Shh is needed for maximal Hk2 induction and to up-regulate the full complement of genes that cooperate with Hk2 for maximal lactate production. In contrast, hypoxia alone induced Hk2 and near-maximal glycolysis in the absence of Shh and insulin. Insulin without Shh did not augment the induction of Hk2 by hypoxia. These findings demonstrate that Shh and N2 acted interdependently to induce glycolysis during cerebellar development, through a mechanism distinct from the induction of glycolysis by hypoxia. 

Previous investigation demonstrated that Shh and insulin/IGF/PI3K signaling pathways converge in CGNPs to activate N-myc [14]. We therefore tested whether N-myc activity mediated developmental induction of Hk2. To disrupt N-myc activity and block potential complementation through C-myc, we treated CGNPs cultured with Shh and N2 with the Myc inhibitor 10058-F4 [26], which blocks association with Max, an interaction required for Myc transcriptional regulation [27]. We then compared expression of Hk2 with that of Cyclin D2, a marker of Shh-induced proliferation, and Cip2A, a known myc target previously demonstrated to be inhibited by 10058-F4 [28,29]. We found 10058-F4 reduced Shh-mediated induction of Hk2, Cip2A and Cyclin D2 in a dose-dependent manner (Figure 4C). These data implicate myc–max transcriptional activation in the induction of Hk2 by the combination of Shh and PI3K signaling.

### Hk2 is required for Shh-induced aerobic glycolysis

**Figure 5:**
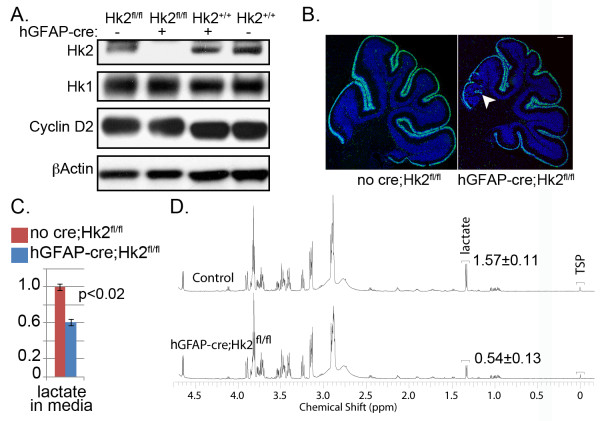
**Conditional deletion of Hk2 prevents Shh-induced aerobic glycolysis. (A)** Representative Western blot comparing Hk2, Hk1 and Cyclin D2 expression in cerebellar lysates at P5 from Hk2^fl/fl^ and Hk2^+/+^ mice with and without hGFAP-cre. Equivalent results were obtained in 5 pups for each genotype at each age. **(B)** EdU incorporation (green) 24 hours after IP injection demonstrates proliferation in the EGL of Hk2^fl/fl^ mice without cre (control) or of hGFAP-cre;Hk2^fl/fl^ mice. White arrowhead marks focus of ectopic proliferation. Nuclei are counterstained with DAPI. Scale bars = 100 μm. **(C)** Lactate accumulation in media from hGFAP-cre;Hk2^fl/fl^ CGNPs was markedly reduced compared with CGNPs of Hk2^fl/fl^ mice without cre. Data compiled from 3 replicates per condition and mean ± SEM values are presented as fold-change relative to control without cre. Two-tailed Student’s *t* test was used for statistical comparison. **(D)** Representative NMR spectra from media samples of Hk2^fl/fl^ mice without cre (control) or of hGFAP-cre;Hk2^fl/fl^ mice. Experiment performed with 3 replicates and mean ± SEM values presented. Lactate was the only detectable metabolite to be significantly altered by Hk2 deletion (*P* < 0.02, two-way analysis of variance with Bonferroni correction).

To test for a causal link between Shh-induced Hk2 expression and aerobic glycolysis, we examined CGNP metabolism in Hk2 conditional knockout mice. We crossed the Hk2-floxed (Hk2^fl/fl^) mouse line EM:02074 with a hGFAP-cre line that drives cre-mediated recombination in embryonic cerebellar stem cells [30]. We chose this specific cre driver because of the high rate of tumorigenesis when combined with the SmoM2 allele, as described below [31]. The hGFAP-cre;Hk2^fl/fl^ mice were obtained at expected Mendelian ratios and were fertile without overt deficits. Western blot analysis of cerebellar lysates at P7 demonstrated up-regulation of Hk2 equivalent to wildtype mice in either the hGFAP-cre or the Hk2^fl/fl^ genotypes, and showed an absence of Hk2 protein in the hGFAP-cre;Hk2^fl/fl^ genotype (Figure 5A). We found no change in Hk1 or Cyclin D2 expression that correlated with Hk2 deletion (Figure 5A). Consistent with the lack of change in Cyclin D2 levels, EdU labeling confirmed that deletion of Hk2 did not markedly reduce CGNP proliferation (Figure 5B). EdU imaging did, however, reveal focal disorganization of the EGL (Figure 5B), as discussed further below. 

Importantly, increased glycolysis in response to Shh was significantly reduced in CGNPs from hGFAP-cre;Hk2^fl/fl^ mice. We compared lactate production in Shh-treated CGNPs isolated either from hGFAP-cre;Hk2^fl/fl^ mice or from littermate Hk2^fl/fl^ controls that lacked the hGFAP-cre transgene. We found that while Hk2-deficient CGNPs proliferated in response to Shh, they generated 40% less lactate than CGNPs from littermates with intact Hk2 (Figure 5C). NMR analysis of media samples also demonstrated decreased lactate production (Figure 5D). Orthogonal partial least squares discriminant analysis identified lactate as the only metabolite to change significantly with deletion of Hk2; a trend toward decreased glucose utilization with Hk2 deletion was also detected, but this change was not statistically significant relative to the overall glucose concentration (data not shown). Genetic deletion of Hk2 thus reduced glycolysis without causing CGNPs to catabolize alternative energy substrates, consistent with increasing efficiency of glucose utilization through oxidative phosphorylation.

### Deletion of Hk2 disrupts CGNP development

**Figure 6:**
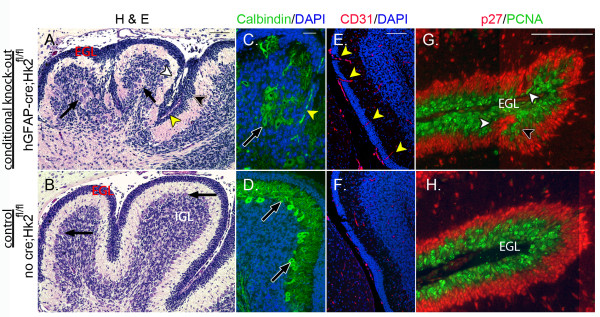
**Hk2 deletion causes focal disruption of cerebellar development.** Hk2 deletion caused focal disruption of cerebellar development as shown by comparison of cerebella with Hk2 deletion (top row) or with intact Hk2 (bottom row). Representative H & E-stained sections demonstrate focal disorganization of the CGNP lineage in hGFAP-cre;Hk2^fl/fl^ mice **(A)**, with regions of focal thickening (black arrowhead) and thinning (white arrowhead) and regions in which CGNPs failed to migrate over Purkinje cells (PC; black arrows) that were inwardly displaced. Yellow arrowhead, ectopic capillary in the EGL. By contrast, the EGL was evenly layered in an identical region of cerebellum of a Hk2^fl/fl^ mouse without cre **(B)** and CGNPs completed migration across the PC layer (black arrows) to the IGL. **(C)** IHC for PC marker Calbindin (green) demonstrates a collection of PCs surrounded by the IGL in a hGFAP-cre;Hk2^fl/fl^ mouse. Also note the presence of an ectopic capillary (yellow arrowhead), containing green autofluorescent red blood cells. **(D)** The IGL formed appropriately inside the PC layer in Hk2^fl/fl^ mice without cre. **(E,F)** IHC for endothelial marker CD31 (red) demonstrates interruption of Hk2-deficient EGL by capillaries (yellow arrowheads). Nuclei are counterstained with DAPI. **(G, H)** IHC for PCNA (green) demonstrates the external, proliferative region of the EGL, while IHC for p27 (red) demonstrates the internal region of the EGL where CGNPs exit the cell cycle and begin to differentiate. The regular separation of layers within the EGL is disrupted in Hk2-deficient cerebella, with focal thinning (white arrowheads) and thickening of the proliferative PCNA^+^ layer (black arrowhead) and complementary change to the p27^+^ layer. All scale bars = 100 μm.

Although proliferation did not appear to be reduced by Hk2 deletion, examination of P7 cerebella from hGFAP-cre;Hk2^fl/fl^ mice revealed focal regions of disorganization within the grossly normal cerebellar architecture. While the EGL at P7 is typically highly regular in thickness, in hGFAP-cre;Hk2^fl/fl^ mice we noted discrete regions of focal thinning or thickening, abnormal migration, and increased vascularization (Figures 5B and 6A to 6H). CGNPs frequently failed to migrate over the Purkinje cell layer as expected, and instead accumulated on both sides of the Purkinje cells (Figure 6A to 6D) and at times divided and displaced portions the EGL, as in Figure 5B. Importantly, small blood vessels, highlighted by CD31 expression, interrupted the EGL of hGFAP-cre;Hk2^fl/fl^ mice (Figure 6E,F). Within the EGL, deletion of Hk2 disrupted the expected correspondence between radial position and differentiation. The EGL is typically comprised of an outer region of proliferating cells that express PCNA and an inner region of PCNA-negative cells in the earliest stage of differentiation, marked by up-regulation of p27 [32]. In the EGL of hGFAP-cre;Hk2^fl/fl^ mice, however, CGNPs failed to maintain the expected correspondence between radial position and expression of either PCNA or p27 (Figure 6G,H). Therefore, although motor impairments were not detected in hGFAP-cre;Hk2^fl/fl^ mice, genetic deletion of Hk2 altered the migration, differentiation and vascularization of progenitors active during postnatal cerebellar development. 

### Deletion of Hk2 disrupts medulloblastoma growth

**Figure 7:**
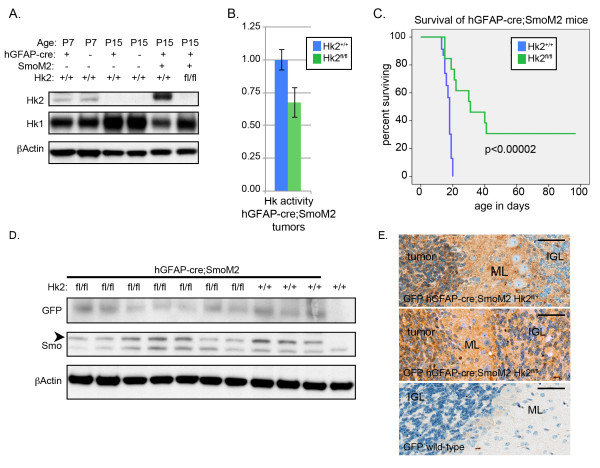
**Hk2 deletion blunts aggressiveness of hGFAP-cre;SmoM2-driven medulloblastoma and extends survival. (A)** Western blot comparing expression of Hk2 and Hk1 in mice with indicated genotype at P7 and P15. Expression of Hk2 protein in SmoM2 tumors with Hk2^+/+^ genotype exceeded the expression of Hk2 in wildtype mice at P7. Hk2 protein was absent in SmoM2 tumors with the Hk2^fl/fl^ genotype. **(B)** Comparison of total Hk activity in tumors with the Hk2^+/+^ or Hk2^fl/fl^ genotype. Two-tailed Student’s *t* test used for statistical comparison. **(C)** Kaplan–Meier curves demonstrate increased event-free survival in hGFAP-cre;SmoM2;Hk2^fl/fl^ mice, compared with hGFAP-cre;SmoM2;Hk2^+/+^ mice (*P* < 0.00002, log-rank test). While 100% (24/24) of hGFAP-cre;SmoM2;Hk2^+/+^ mice developed symptomatic tumor requiring euthanasia by P20, 30% (4/13) of hGFAP-cre;SmoM2;Hk2^fl/fl^ mice remained alive without symptoms at P100. **(D)** Western blot for GFP and Smoothened (Smo) demonstrates expression of SmoM2-YFP fusion protein (arrowhead) in hGFAP-cre;SmoM2 mice with either the Hk^fl/fl^ or the Hk2^+/+^ genotype. The SmoM2 allele is detected in the Smoothened blot as a band (arrowhead) that is not present in wildtype P8 cerebella and is of higher molecular weight than the wildtype protein. **(E)** IHC for GFP (brown) demonstrates SmoM2-YFP protein in the neoplastic and differentiated regions of cerebella from hGFAP-cre;SmoM2 mice with either the Hk^fl/+^ genotype (upper panel) or the Hk2 ^fl/fl^ genotype (middle panel). A section of cerebella from a wildtype mouse, processed in parallel, demonstrated absence of staining with GFP antibody under identical conditions (lower panel). Nuclei are counterstained blue with hematoxylin. Scale bars = 50 μm.

To determine how loss of Hk2 and anticipated disruption of aerobic glycolysis would impact medulloblastoma, we bred hGFAP-cre;Hk2^fl/fl^ mice with cre-inducible SmoM2 mice [13] to derive medulloblastoma-prone hGFAP-cre;SmoM2 mice with Hk2^+/+^, Hk2^fl/+^ or Hk2^fl/fl^ genotypes. Activation of SmoM2 by hGFAP-cre induced robust tumorigenesis: 100% of hGFAP-cre;SmoM2;Hk2^+/+^ mice developed grossly visible occipital expansion by P12. At P12, no mice demonstrated neurologic deterioration. Over the following days, however, mice developed discernible neurologic symptoms such that by P20 100% of mice required euthanasia due to symptomatic medulloblastoma. Median survival was 18 days (Figure 7C) with no sex-linked variation (data not shown). Western blot analysis demonstrated that while hGFAP-cre and wildtype mice down-regulated Hk2 by P15, Hk2 was highly expressed at P15 in hGFAP-cre;SmoM2 Hk2^+/+^ tumors. Hk2 was in fact significantly more abundant in SmoM2 tumors than in P7 cerebella from either wildtype or GFAP-cre mice (Figure 7A). Hk2 protein was absent, however, from tumors in hGFAP-cre;Hk2^fl/fl^ mice. Total Hk activity was markedly reduced by Hk2 deletion (Figure 7B). 

In contrast to the rapidly progressive Hk2 wildtype tumors, medulloblastomas with Hk2 deletion were markedly less malignant (Figure 7C). While 100% of P12 hGFAP-cre;SmoM2;Hk2^fl/fl^ mice developed the characteristic occipital expansion that is the first sign of tumor, median survival was 31 days (*P* < 0.00002) and 30% survived event-free to the end of the experiment at 100 days. Importantly, these long-term survivors were able to breed with wildtype mice to give rise to hGFAP-cre;SmoM2;Hk2^fl/+^ progeny that developed malignant tumors, demonstrating the efficacy of the inherited SmoM2 and hGFAP-cre alleles. All hGFAP-cre;SmoM2;Hk2^fl/+^ mice, like hGFAP-cre;SmoM2;Hk2^+/+^ mice, died by P20 (data not shown). Western blot analysis demonstrated expression of the SmoM2-YFP fusion protein in both hGFAP;SmoM2;Hk2^+/+^ and hGFAP;SmoM2;Hk2^fl/fl^ mice (Figure 7D), while IHC for GFP revealed SmoM2-YFP expression throughout the cerebella of hGFAP-cre; SmoM2 mice, both in tumor and in adjacent, differentiated regions, in both the Hk2^+/+^ and Hk2^f/fl^ genotypes (Figure 7E). Mosaicism for SmoM2 was not observed in any GFP-stained sections. Mice with the hGFAP-cre;SmoM2;Hk2^fl/fl^ genotype thus expressed SmoM2 and developed tumors, but these tumors progressed less rapidly than tumors with at least one functional allele of Hk2.

**Figure 8:**
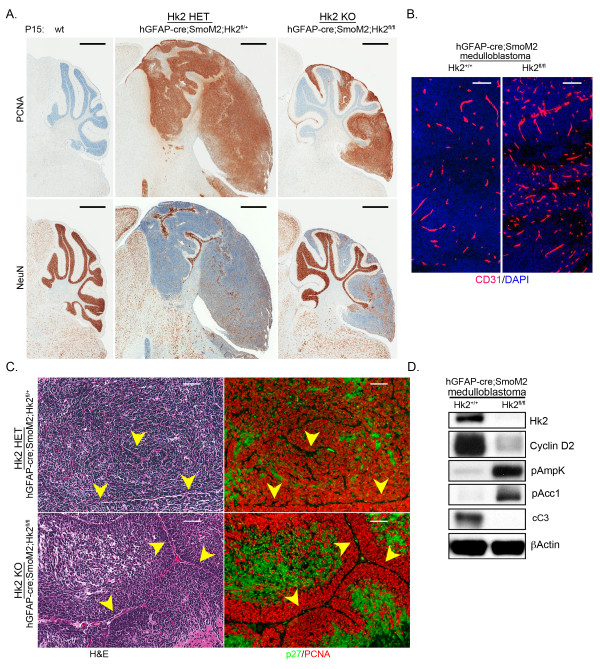
**Deletion of Hk2 caused specific changes in pathology and pathway activation in hGFAP-cre;SmoM2-driven medulloblastoma.(A)** Comparison of cerebella at P15 from wildtype (wt; left column), hGFAP-cre;SmoM2;Hk2^fl/+^ (middle column) and hGFAP-cre;SmoM2;Hk2^fl/fl^ (right column) mice. Proliferating cells were visualized by IHC for PCNA (top row) and differentiated neurons were labeled by IHC for NeuN (bottom row). Antibodies are visualized in brown, and nuclei are counterstained blue with hematoxylin. At least 3 tumors of each genotype were examined and representative images are presented. **(B)** IHC for endothelial marker CD31 (red) demonstrates increased capillary density in Hk2-deficient medulloblastoma. Nuclei are counterstained with DAPI. **(C)** Comparison of proliferation and early differentiation using IHC for PCNA (green) and p27 (red) respectively, in hGFAP-cre;SmoM2;Hk2^fl/+^ (top row) or hGFAP-cre;SmoM2;Hk2^fl/fl^ (bottom row) medulloblastoma. H & E-stained sections are provided for reference (left column). Yellow arrowheads highlight blood vessels. Proliferating tumor cells in Hk2^fl/fl^ medulloblastoma concentrated around blood vessels, in contrast to the even distribution of proliferating cells in Hk2^fl/+^ tumors. In Hk2^fl/fl^ medulloblastoma, tumor cells that were further from the perivascular region were PCNA^–^ and p27^+^, indicating cell cycle exit. **(D)** Western blot demonstrates increased phosphorylation of AMP-activated kinase (AMPk) and Acyl-CoA Carboxylase (Acc1) in Hk2^fl/fl^ medulloblastoma, along with reduced expression of proliferation marker Cyclin D2. Decreased abundance of cC3 in Hk2^fl/fl^ medulloblastoma demonstrates that loss of Hk2 did not induce apoptosis. Scale bars = 1,000 μm (**A**), 100 μm (**B**) and 50 μm (**C**).

Along with increasing survival time, deletion of Hk2 profoundly altered tumor pathology. As expected, proliferation was minimal in wildtype cerebella at P15, where CGNPs had completed migration to the IGL and terminal differentiation into NeuN^+^ neurons (Figure 8A, left column). In 100% of hGFAP-cre;SmoM2;Hk2^+/+^ or Hk2^fl/+^ mice, however, the entire posterior fossa was filled with PCNA^+^ tumor cells in an expanded EGL by P15; a relatively small fraction of CGNPs migrated to the IGL, ceased proliferation and expressed NeuN (Figure 8A, middle column). In contrast, hGFAP-cre;SmoM2;Hk2^fl/fl^ mice demonstrated a smaller PCNA^+^ population in the EGL and a markedly larger proportion of CGNPs that differentiated to become NeuN^+^ CGNs in the IGL (Figure 8A, right column). Therefore, while deletion of Hk2 did not block proliferation, the deletion did reduce the sustained proliferation caused by SmoM2, as SmoM2-expressing CGNPs with Hk2 deletion exited the cycle in vastly greater numbers. Importantly, progenitors in hGFAP-cre;SmoM2;Hk2^fl/fl^ mice that exited the cell cycle proceeded with the migration and terminal differentiation typical of the CGNP lineage, generating a relatively normal cerebellar architecture.

### Hk2 links energy metabolism and maintenance of an undifferentiated state

Along with increased differentiation, Hk2-deficient tumors demonstrated increased micro-vascularization (Figure 8B) and increased localization of proliferative cells along blood vessels (Figure 8C). Between regions of perivascular proliferation, tumor cells exited the cell cycle and up-regulated the early differentiation marker p27 (Figure 8C). The close correlation between proliferation and distance from capillaries suggested that sustained proliferation in Hk2-deficient tumors might depend on availability of oxygen. Accordingly, reduced tumor growth might result from inability to meet energy needs outside the perivascular region.

To probe for a link between impaired energy production and reduced tumor growth in Hk2-deficient medulloblastoma, we compared phosphorylation of AMPk in cerebella from hGFAP-cre;SmoM2;Hk2^+/+^ mice and hGFAP-cre;SmoM2;Hk2^fl/fl^ mice. At P15, cerebella from both genotypes expressed SmoM2, but hGFAP-cre;SmoM2;Hk2^+/+^ cerebella were almost entirely replaced by tumor while hGFAP-cre;SmoM2;Hk2^fl/fl^ cerebella contained both tumor and differentiated tissue. We integrated the AMPk results with a comparison of proliferation and apoptosis by Western blot analysis for specific markers (Figure 8F). AMPk functions as an intracellular energy sensor, becoming phosphorylated in response to cellular energy scarcity [33]. In Hk2-deficient medulloblastomas, phosphorylation of AMPk was greatly increased, consistent with energy scarcity. Phosphorylation of Acc1, a known target of AMPk [34], was also increased, indicating that the detected increase in AMPk activity was functionally relevant. We examined cC3 because we have previously found that medulloblastomas demonstrate continuous baseline apoptosis demonstrable with cC3 staining [35]. Interestingly, both Cyclin D2 and cC3 were markedly less abundant in Hk2^fl/fl^ medulloblastomas, demonstrating that while energy scarcity correlated with reduced proliferation, it did not provoke cell death. Deletion of Hk2 thus reduced the ability of progenitors to remain undifferentiated without altering survival. Taken together, these findings support a model in which: 1) aerobic glycolysis supports the undifferentiated progenitor phenotype of medulloblastoma by preserving energy homeostasis; and 2) impaired energy homeostasis caused by Hk2 deletion promotes differentiation, both disrupting patterning in development and reducing tumor growth.

## Discussion

Since its initial observation by Otto Warburg, aerobic glycolysis has been documented in a variety of cancer cells and in non-neoplastic thymocytes [36]. The selective advantage of aerobic glycolysis in cancer has been directly tested by comparing growth rates of xenograft tumors with and without PkM2 [37] or Hk2 [4]. Why cells should harbor a genetic program that promotes cancer growth, however, has been unclear. Our data demonstrate that aerobic glycolysis is a developmental program that is co-opted in the course of *in vivo* tumorigenic transformation. Importantly, we identified Hk2 as a key mechanism through which developmental signaling molecules induce aerobic glycolysis. Moreover, the Hk2-dependent, glycolytic metabolism of mitotic CGNPs was maintained in medulloblastoma and was essential to cancer pathogenesis: disrupting glycolysis through conditional deletion of Hk2 markedly reduced tumor growth while increasing differentiation. These findings reveal cancer cells exploiting the specialized energy metabolism of developmental progenitors to maintain an undifferentiated state and malignant potential.

We noted that Hk2 up-regulation exerted profound effects on glucose metabolism in CGNPs that exceeded the change in Hk activity measured in lysates. Our data indicate that Hk2 is required for maximal glycolysis, and functions optimally to shunt glucose toward glycolysis in the intact cell. Hk2 is known to localize to the outer mitochondrial membrane, and this subcellular localization is critical to its pro-glycolytic effect [38]. Accordingly, it is not surprising that the measured effect of increased Hk2 in lysates underestimates the influence of Hk2 on glucose metabolism in live cells.

The nature of the benefit that glycolysis confers on dividing cells is controversial. Since oxidative phosphorylation generates more ATP per glucose molecule, an open question is why cells metabolize any glucose to lactate when oxygen is not limiting [39]. Potential cellular benefits of aerobic glycolysis for mitotic cells may include supplying intermediaries for lipid and nucleic acid synthesis [1], or enabling high glucose flux [39]. High glucose flux has been observed to exert an anti-apoptotic effect through diverse mechanisms including Bax inactivation [40], prevention of cytochrome *c* release [41], or redox inactivation of cytochrome *c*[42]. Our findings that genetic deletion of Hk2 disrupted both energy homeostasis and the balance between proliferation and differentiation provide new insight into the question of how aerobic glycolysis can support progenitor function.

A key aspect of the progenitor state is the maintenance of self-renewal capacity that allows daughter cells to remain proliferative after cell division. In the course of developmental CGNP proliferation, the capacity for self-renewal is gradually lost and the CGNP population declines until proliferation ceases. In contrast, under the influence of SmoM2-driven tumorigenesis, self-renewal is maintained and the EGL grows unconstrained. Hk2 deletion disrupted the maintenance of self-renewal capacity, causing premature differentiation of CGNPs in the EGL, manifested as focal disorganization. In medulloblastoma, however, where self-renewal capacity does not typically wane, Hk2 deletion caused a much greater effect, disrupting unconstrained growth, promoting increased differentiation and increasing the probability of animal survival. The abnormal migration patterns of Hk2-deleted CGNPs and the reduced growth of Hk2-deleted tumors thus consistently highlight a role for Hk2-driven glycolysis in maintaining the undifferentiated progenitor state.

The increased vascularity of Hk2-deleted EGL and medulloblastoma implicate oxygen homeostasis in progenitor function. By up-regulating Hk2-dependent glycolysis, wildtype CGNPs reduce oxygen dependence at the cost of increased utilization of glucose (Figure 1). By blocking Shh-driven glycolysis, Hk2 deletion increased the dependence of CGNPs and medulloblastoma on vascular support. The ectopic capillaries formed in the Hk2-deficient EGL(Figure 6) and the tumor cells in Hk2^fl/fl^ medulloblastoma that proliferated along capillaries (Figure 7), both demonstrated increased vascular dependence caused by loss of Hk2. In Hk2-deficient cerebella, increased vascularity effectively compensated for the loss of aerobic glycolysis and cerebellar development was largely preserved despite local failures of migration and differentiation. In Hk2-deficient medulloblastomas, however, increased vascularization was insufficient to compensate, perhaps due to the increased tumor mass or alternatively due to increased metabolic demand. Importantly, vascular compensation did not prevent cellular energy scarcity, as demonstrated by activation of AMPk. Therefore, with increased vessel support, oxidative phosphorylation could meet the energy needs of developmentally regulated neurogenesis, but not of unconstrained tumor growth. Importantly, the consequence of energy failure in medulloblastoma was a loss of progenitor state and progression through developmentally appropriate differentiation. Similarly, recent investigation has demonstrated that AMPk activation blocks the reprogramming of mouse embryonic fibroblasts into induced stem cells [43]; in each case, activation of AMPk blocks the maintenance of progenitor self-renewal and promotes terminal differentiation.

An important benefit of aerobic glycolysis for CGNPs may be to prevent energy failure that could limit proliferative potential. Neural stem cells of the forebrain and hippocampus are known to require the support of a perivascular niche [44-47]. In contrast to these discrete sites of postnatal neurogenesis, the EGL, where CGNPs proliferate, extends over a broad area; to achieve even growth throughout the EGL, CGNPs must proliferate at a constant rate regardless of distance from the supportive niche. Aerobic glycolysis, through reduced oxygen dependence at the cost of increased glucose dependence, may reduce the need for perivascular support. While oxygen must be obtained from capillaries, glucose can be mobilized from intracellular stores or provided by neighboring cells through active transport. Aerobic glycolysis may thus release progenitors from the perivascular niche, and disrupting Hk2 may inhibit tumorigenesis by constraining progenitors to regions of niche support.

Consistent with an integral role in neurogenesis, we found aerobic glycolysis to be regulated by developmental signaling. In particular, we found that induction of glycolysis requires co-incident activation of Shh and insulin/IGF/PI3K pathways. Importantly, we identified Myc–Max-mediated transcriptional regulation as a specific downstream effector necessary for this regulation. Such a role, would be consistent with previous investigations that have linked c-myc to the Warburg effect in other cell types [48,49]. Modulation of intracellular calcium has also been identified as a point of convergence for Shh and PI3K signaling [50] and could play a key role in mediating Shh effects on CGNP glycolysis. Alongside our finding that Shh regulates carbohydrate metabolism, recently published work has demonstrated that Shh down-regulates fatty acid oxidation in favor of lipid biosynthesis [51]. Importantly, we found that blockade of Shh pathway-induced glycolysis in medulloblastoma caused both reduced proliferation and inactivation of Acc1, a critical regulator of lipid metabolism. These findings together define a mitogen-induced metabolic configuration in which carbohydrate and lipid metabolism are integrated to optimally support progenitor proliferation.

## Conclusions

Our results demonstrate that aerobic glycolysis is primarily a neurodevelopmental program that is co-opted in medulloblastoma tumorigenesis to promote neoplastic growth. We found that medulloblastoma and neural progenitors share specific metabolic requirements that distinguish them from post-mitotic brain cells. Our results showing increased survival in medulloblastoma-bearing mice with Hk2 deletion have revealed an unexpected link between cellular metabolism and differentiation state. In Hk2-deleted tumors where Shh-driven glycolysis was prevented, the consequence was increased differentiation and reduced tumor growth. Together, these findings connect developmental signaling pathways with patterns of metabolism in cancer while also demonstrating the potential efficacy of metabolic therapy for medulloblastoma through targeting of Hk2.

## Abbreviations

Acc1: AcylCoA Carboxylase; AMPk: AMP-activated kinase; cC3: Cleaved caspase-3; CGN: Cerebellar granule neuron; CGNP: Cerebellar granule neuron progenitor; DAPI: 4^′^6-diamino-2-phenylindole; CT: Computed tomography; DMEM: Dulbecco’s modified Eagle’s medium; EGL: External granule cell layer; FCCP: Trifluorocarbonylcyanide phenylhydrazone; FCS: Fetal calf serum; FDG: Fluorodeoxiglucose; GFP: Green fluorescent protein; H & E: Hematoxylin and eosin; Hk: Hexokinase; IGF: Insulin-like growth factor; IGL: Internal granule cell layer; IHC: Immunohistochemistry; IP: Intraperitoneal; MRS: Magnetic resonance spectroscopy; NMR: Nuclear magnetic resonance; OCR: Oxygen consumption rate; P: Postnatal day; PBS: Phosphate-buffered saline; PCNA: Proliferating cell nuclear antigen; PCR: Polymerase chain reaction; PET: Positron emission tomography; PI3K: Phosphoinositide 3-kinase; RT: Reverse transcriptase; Shh: Sonic Hedgehog.

## Competing interests

The authors declare that they have no competing interests.

## Authors’ contributions

TRG conceived of the study, participated in its design and coordination, and drafted the manuscript. AJC carried out cell culture and mouse experiments and helped draft the manuscript. IG assisted in cell culture experiments and editing of the manuscript. RA performed OCR studies. HY conducted ^18^FDG experiments and small animal imaging. CRM provided neuropathology expertise and editorial input. AT and JM conducted NMR studies and metabolomic analysis. JO participated in study design, interpretation of data, and drafting of manuscript. MD provided key input in study design, interpretation of data, drafting of manuscript. All authors read and approved the final manuscript.

## Supplementary Material

Additional file 1 **Figure S1.** Induction of lactate production by Shh did not depend on high glucose or KCl concentration. Comparison of media lactate concentrations measured by the colorimetric method, from CGNPs cultured in DMEM/F12, supplemented with N2 plus vehicle or Shh, and KCl as indicated, and from CGNPs cultured in indicated typical or low-glucose media, supplemented with N2 and either vehicle or Shh. Click here for file
