# Supplementary figures and images for: Hexokinase-2-mediated aerobic glycolysis is integral to cerebellar neurogenesis and pathogenesis of medulloblastoma
Source: Cancer Metab. 2013 Jan 23;1:2. doi: 10.1186/2049-3002-1-2 (PMC3782751; doi:10.1186/2049-3002-1-2)

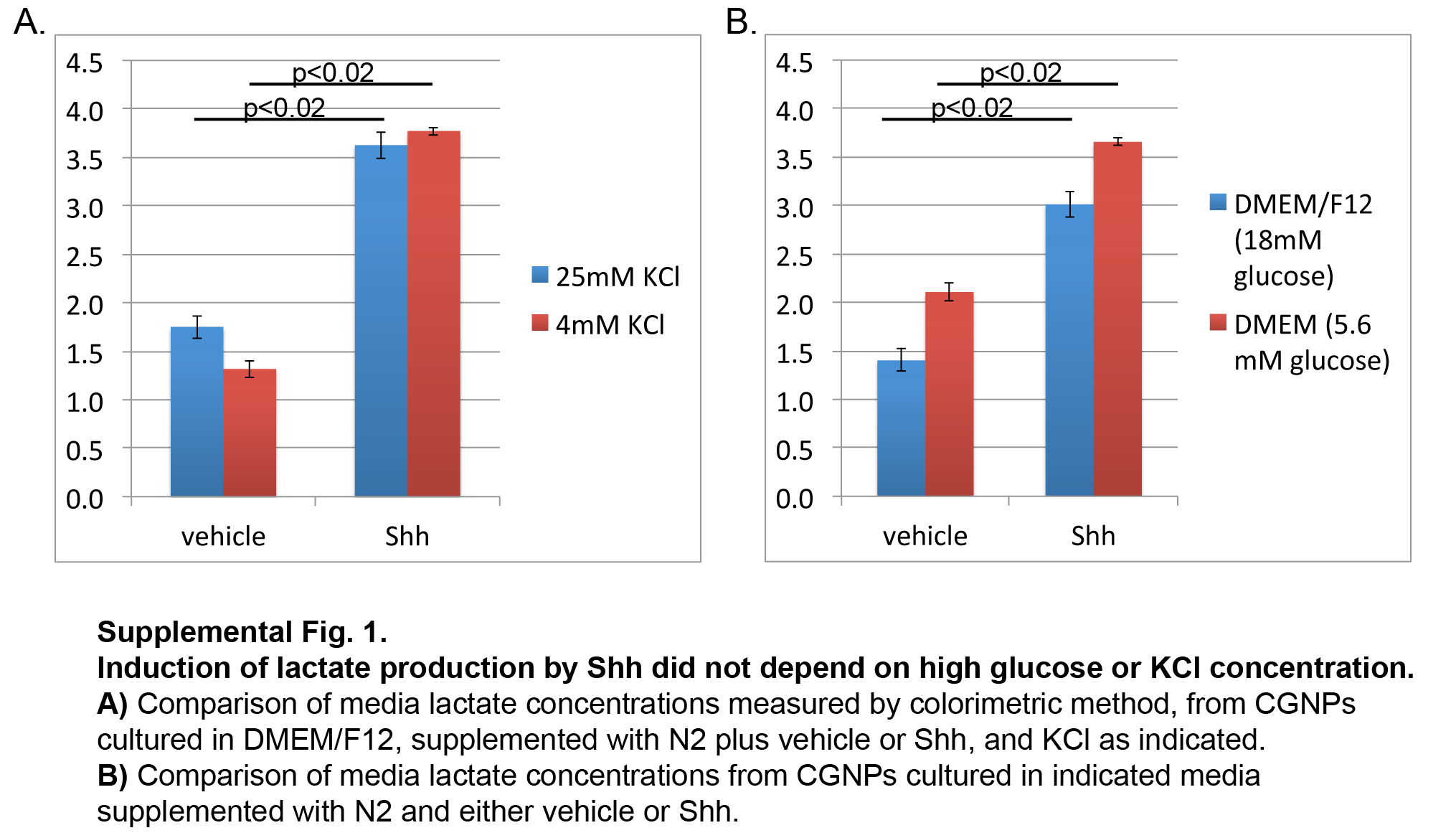

Supplement: Additional file 1 — Figure S1. Induction of lactate production by Shh did not depend on high glucose or KCl concentration. Comparison of media lactate concentrations measured by the colorimetric method, from CGNPs cultured in DMEM/F12, supplemented with N2 plus vehicle or Shh, and KCl as indicated, and from CGNPs cultured in indicated typical or low-glucose media, supplemented with N2 and either vehicle or Shh. [file 2049-3002-1-2-S1.tiff]
